# Increased Incidence of *Campylobacter* spp. Infection and High Rates among Children, Israel

**DOI:** 10.3201/eid1911.120900

**Published:** 2013-11

**Authors:** Miriam Weinberger, Larisa Lerner, Lea Valinsky, Jacob Moran-Gilad, Israel Nissan, Vered Agmon, Chava Peretz

**Affiliations:** Assaf Harofeh Medical Center, Zerifin, Israel (M. Weinberger);; Tel Aviv University Sackler Faculty of Medicine, Tel Aviv, Israel (M. Weinberger, C. Peretz);; Israel Ministry of Health, Jerusalem, Israel (L. Lerman, L. Valinsky, I. Nissan, V. Agmon);; Israel Ministry of Health Public Health Services, Jerusalem (J. Moran-Gilad)

**Keywords:** Campylobacter, Campylobacter jejuni, Campylobacter coli, foodborne illnesses, incidence, Poisson distribution, Israel, epidemiology, children, pediatric, campylobacteriosis, bacteria, enteric infections

## Abstract

During 1999–2010, the annual incidence of *Campylobacter* spp. infection in Israel increased from 31.04 to 90.99 cases/100,000 population, a yearly increase of 10.24%. Children <2 years of age were disproportionally affected; incidence in this age group (356.12 cases/100,000 population) was >26-fold higher than for the 30–<50 age group.

*Campylobacter* spp. have become the leading cause of foodborne infections in many industrialized countries, despite extensive control efforts (*1*). Recent studies suggest that *Campylobacter* spp. infection in Israel may also be on the rise (*2*), in contrast to a substantial decrease in the incidence of *Salmonella* spp. infection, from 86.9 cases/100,000 population in 1995 to 44.0 cases/100,000 population in 2009 (*3*). We examined recent trends of *Campylobacter* spp. infection in Israel, with a focus on age- and sex-specific rates of infection.

## The Study

Campylobacteriosis is a reportable disease in Israel. Microbiology laboratories countrywide passively submit human isolates from all sources to the National *Campylobacter* Reference Laboratory, Israeli Ministry of Health, Jerusalem, for confirmatory testing. Species are identified by using standard methods (*4*). The reporting system and laboratory methods did not change during the study period of January 1, 1999–December 31, 2010.

For this study, patients’ date of birth and sex were retrieved using special permission by using identification numbers, which were subsequently replaced by unique numbers to retain patient anonymity. An infection episode was defined as the isolation of *Campylobacter* spp. from a single patient from any clinical source. Annual incidence rates for the study period were calculated by dividing the number of annual infection episodes by the population size retrieved from the Israeli Bureau of Statistics (*5*). The average age-specific annual incidence rate was calculated on the basis of the 12 annual incidence rates obtained for the study period. Because incidence counts and rates follow a Poisson distribution, Poisson regression models accounting for overdispersion were used to study annual trends of the incidence rate (dependent variable) for all isolates and for 2 major *Campylobacter* species, *C. jejuni* and *C. coli*; the calendar year was the independent variable. Poisson models were also used to study the effects of sex and age group on the incidence rates, adjusted for annual trends. All model effects were expressed by incidence rate ratio (IRR) and 95% CI. SAS software version 9.2 (SAS Institute, Cary, NC, USA) was used for all analyses. The study was approved by the Assaf Harofeh Medical Center local ethics committee.

During the study period, the *Campylobacter* Reference Laboratory confirmed 47,253 episodes of *Campylobacter* spp. infection. Most (>99%) infections were *C. jejuni* (37,062 episodes, 78.43%) and *C. coli* (10,092 episodes, 21.36%); the remaining <1% were *C. fetus* (25 episodes), *C. upsaliensis* (6 episodes), *C. lari* (2 episodes), or unidentified species (66 episodes). Bacteremia was noted for 331 (0.7%) episodes.

During the 12 study years, the annual incidence rate of all laboratory-confirmed *Campylobacter* spp. infection episodes increased 2.93-fold, from 31.04 to 90.99 cases/100,000 population. A similar increase was observed for *C. jejuni* (2.87-fold, 24.59 to 70.54 cases/100,000) and *C. coli* (3.06-fold, 6.38 to 19.54 cases/100,000). The linear annual increase in the incidence rate for the entire study period was 10.24% (95% CI 8.46–12.06) for all episodes, 10.07% (95% CI 8.42–11.74) for *C. jejuni* episodes, and 10.73% (95% CI 8.19–13.33) for *C. coli* episodes. A sharp rise in the annual increase rate, from 8.22% (95% CI 4.88–11.68) to 18.97% (95% CI 12.95–25.31), was noted between 1999–2006 and 2007–2010 (period I and period II) (Table).

**Table:** Incidence of *Campylobacte*r spp. infection by study period, sex, and age group, Israel 1999–2010

*Campylobacter* species	Incidence rate ratio (95% CI)		
	Full study period, 1999–2010	Study period I, 1999–2006	Study period II, 2007–2010
All *Campylobacter* spp.			
Annual trend*	1.10 (1.08–1.12)	1.08 (1.05–1.12)	1.19 (1.12–1.25)
Male sex†	1.36 (1.22–1.52)	1.37 (1.18–1.60)	1.35 (1.21–1.51)
Age group, y‡			
0–<2	26.27 (18.68–36.96)	29.96 (18.96–47.33)	22.81 (13.49–38.59)
2–<10	5.50 (3.91–7.75)	5.86 (3.70- 9.28)	5.17 (3.05–8.75)
10–<30	2.34 (1.57–3.50)	2.29 (1.33–3.92)	2.40 (1.30–4.43)
50–<70	1.42 (0.92–2.20)	1.51 (0.85–2.70)	1.33 (0.67–2.63)
>70	1.81 (1.19–2.74)	1.81 (1.03- 3.16)	1.81 (0.95–3.43)
*C. jejuni*			
Annual trend*	1.10 (1.08- 1.12)	1.09 (1.06–1.12)	1.19 (1.12–1.26)
Male sex†	1.39 (1.25–1.54)	1.40 (1.23–1.61)	1.37 (1.21–1.55)
Age group, y‡			
0–<2	28.42 (19.96–40.46)	31.42 (19.89–49.62)	25.45 (14.36–45.09
2–<10	6.04 (4.24–8.61)	6.29 (3.98–9.95)	5.78 (3.26–10.26)
10–<30	2.50 (1.66–3.77)	2.42 (1.42- 4.13)	2.58 (1.33- 4.99)
50–<70	1.42 (0.90–2.23)	1.40 (0.86- 2.74)	1.30 (0.62–2.75)
>70	1.70 (1.09–2.63)	1.73 (0.99- 3.05)	1.66 (0.81–3.37)
*C. coli*			
Annual trend*	1.11 (1.08–1.13)	1.07 (1.01–1.12)	1.17 (1.12- 1.23)
Male sex†	1.28 (1.10–1.50)	1.27 (0.98–1.65)	1.29 (1.15–1.45)
Age group, y ‡			
0–<2	20.16 (14.04–28.94)	25.57 (14.81–44.14)	15.86 (10.12–24.85)
2–<10	3.99 (2.77–5.73)	4.53 (2.61- 7.86)	3.55 (2.26–5.57)
10–<30	1.91 (1.24–2.95)	1.87 (0.97- 3.64)	1.94 (1.13–3.31)
50–<70	1.42 (0.90–2.25)	1.44 (0.72–2.90)	1.40 (0.79–2.48)
>70	2.09 (1.36–3.22)	2.03 (1.06- 3.91)	2.15 (1.27–3.64)

*Per year, adjusted for study years. †Reference: female ‡Reference: 30–<50 y age group.

**Figure 1:**
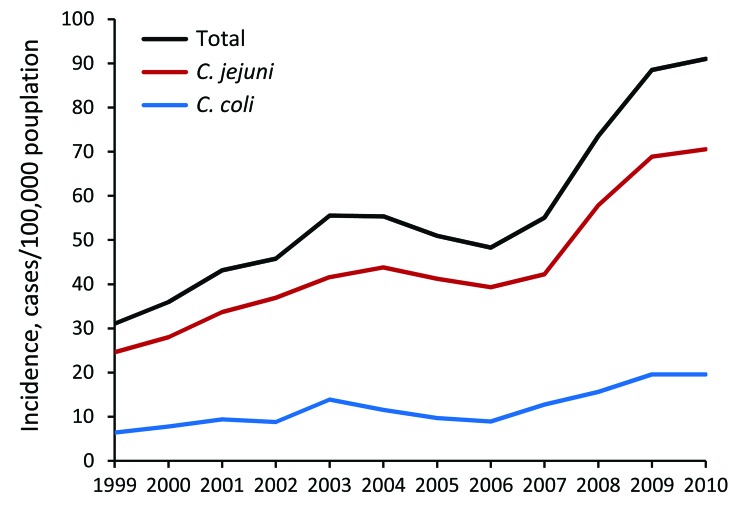
Annual incidence trends of laboratory-confirmed *Campylobacter* spp. infection, by species, Israel, 1999–2010.

Complete patient age and sex data were available for 38,092 (80.63%) of all infection episodes, including 29,931 (80.76%) *C. jejuni* infection episodes and 8,083 (80.09%) *C. coli* infection episodes. The annual incidence trends of *Campylobacter* spp. infection for the subgroup with complete demographic data were similar to those described for the entire group. Further age- and sex-related analyses were completed for episodes for which complete demographic data was available. IRR was 1.36 (95% CI 1.22–1.52) for male sex compared with female sex, adjusted for annual trends; similar elevated rates for male sex were found for *C. jejuni* (IRR 1.39, 95% CI 1.25–1.54) and *C. coli* (IRR 1.28, 95% CI 1.10–1.50) and for the 2 study periods (Figure 1; Table).

The age-specific average annual incidence rate formed an asymmetric, U-shaped curve. The highest average annual incidence rate occurred during the first decade of life (135.44 cases/100,000 population), and more specifically, during the first and second years of life (363.39 and 348.80 cases/100,000 population, respectively). The lowest average annual incidence rate occurred in the fifth decade of life (12.82 cases/100,000 population), with a slight increase toward the eighth decade of life (26.44 cases/100,000 population).

**Figure 2:**
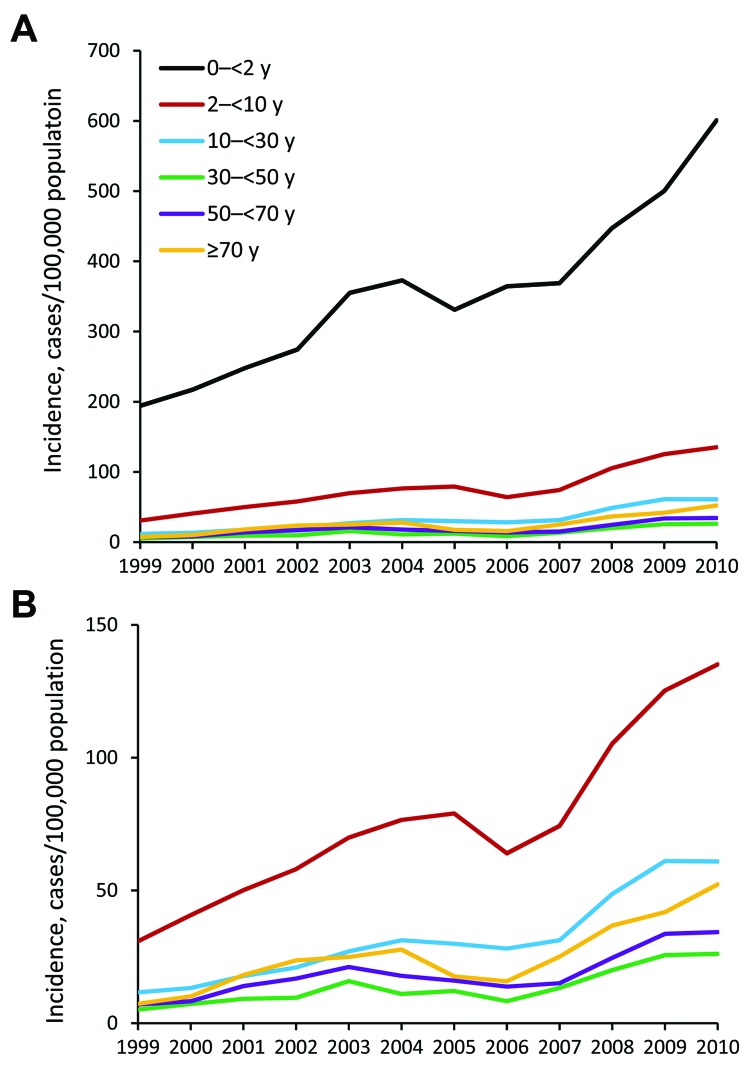
Annual incidence trends of laboratory-confirmed *Campylobacter* infection, by 6 age groups, with (A) and without (B) the very young age group (0–<2 y), Israel, 1999–2010.

Six age groups were established for comparison of incidence rates; the age group of 30–<50 years was used as reference. The average annual incidence rate of infection in age group 0–<2 years (356.12 cases/100,000 population) was 26.27 (95% CI 18.70–36.99) times higher than for the reference group (13.63 cases/100,000 population), adjusted for annual trends. Differences in incidence between the other age groups and the reference age groupwere smaller, ranging from an IRR of 1.42 (95% CI 0.92–2.20) for the 50–<70-year group to an IRR of 5.50 (95% CI 3.91–7.75) for the 2–<10-year age group. Similar IRRs for the respective age groups were found for infection caused by the 2 major *Campylobacter* species and throughout the study periods (Figure 2; Table).

## Conclusions

We found a sharp increase in the incidence of *Campylobacter* spp. infection in Israel, with rates tripling within just 12 years. This trend was observed for the 2 major *Campylobacter* species, *C. jejuni* and *C. coli,* and affected all age groups; the highest infection rates were seen during the first 2 years of life. Infection rates were substantially higher among children <2 years of age compared with rates for other Western countries (*6*,*7*) but were comparable to that reported for New Zealand (*8*). The difference in incidence between this and the other age groups, forming a U-shaped curve, is more characteristic of rates for developing countries and is believed to be indicative of repeated exposure to *Campylobacter* spp. in early childhood that results in the acquisition of protective immunity at older age (*9*). Similar trends have also been described for defined subpopulations in the United Kingdom (*10*).

The global disproportional burden of campylobacteriosis among young children is far from being understood (*11*). A recent study could not show increased exposure to known risk factors in young children compared with other age groups (*12*). Increased susceptibility because of immature immune systems, environmental contamination, cross-contamination in the kitchen, hand-to-mouth behavior, and overreporting have all been implicated.

The rapid increase and high incidence of campylobacteriosis in Israel resemble that of New Zealand (*13*). A food source of *Campylobacter* spp. infection in Israel has not been elucidated; however, during the study period, poultry meat sales markedly transitioned from mainly frozen to mainly fresh or chilled products (S. Dolev, pers. comm.). Similar trends were implicated for the rising incidence in New Zealand and were successfully mitigated by supervising fresh poultry sales (*13*). However, toddlers who do not consume poultry had the highest incidence of *Campylobacter* spp. infection for both countries (*8*).

Our study was conducted using a large and comprehensive national database of laboratory-confirmed *Campylobacter* spp. infections that has a high rate of species characterization. However, laboratory-confirmed infections represent only a small portion of diarrheal diseases (*14*,*15*). Moreover, young children may be more likely to receive medical care and have stool cultured (*14*,*15*).

In conclusion, the rapid increase in *Campylobacter* spp. incidence in Israel illustrates the need for an urgent national intervention plan. In particular, high infection rates among young children should prompt intensive research efforts to discover the routes of exposure*.*
